# Coupling of kinesin ATP turnover to translocation and microtubule regulation: one engine, many machines

**DOI:** 10.1007/s10974-012-9289-6

**Published:** 2012-03-24

**Authors:** Claire T. Friel, Jonathon Howard

**Affiliations:** 1 School of Biomedical Sciences, University of Nottingham, Medical School, Queen’s Medical Centre, Nottingham, NG7 2UH UK; 2 Max Planck Institute of Molecular Cell Biology and Genetics, Pfotenhauerstr. 108, 01307 Dresden, Germany

**Keywords:** Kinesin, ATPase, Microtubule, ATP turnover cycle

## Abstract

The cycle of ATP turnover is integral to the action of motor proteins. Here we discuss how variation in this cycle leads to variation of function observed amongst members of the kinesin superfamily of microtubule associated motor proteins. Variation in the ATP turnover cycle among superfamily members can tune the characteristic kinesin motor to one of the range of microtubule-based functions performed by kinesins. The speed at which ATP is hydrolysed affects the speed of translocation. The ratio of rate constants of ATP turnover in relation to association and dissociation from the microtubule influence the processivity of translocation. Variation in the rate-limiting step of the cycle can reverse the way in which the motor domain interacts with the microtubule producing non-motile kinesins. Because the ATP turnover cycle is not fully understood for the majority of kinesins, much work remains to show how the kinesin engine functions in such a wide variety of molecular machines.

## Introduction

The kinesins are a superfamily of proteins that use the hydrolysis of ATP to regulate their interaction with the microtubule cytoskeleton (Lawrence et al. 2004; Miki et al. 2005; Marx et al. 2009; Kinesin Home Page). The family is defined by a highly conserved motor domain (Marx et al. 2009; Sack et al. 1999). The motor domain acts as a nucleotide-gated switch: its conformation depends on whether ATP, ADP·P_i_, ADP or no nucleotide is bound (Hirose et al. 2006; Kikkawa et al. 2001; Yun et al. 2001). The function of the different conformations is, in part, to regulate the affinity of the motor domain for the microtubule (Hackney 1994; Crevel et al. 1999; Ma and Taylor 1997). The motor domain switches between states of low and high affinity for the microtubule (the so-called strongly bound and weakly bound states) as nucleotide binds, is hydrolyzed and the products released. In turn, binding of the motor domain to the microtubule influences the kinetics of changes in the nucleotide state and therefore the transitions between the different conformational states of the motor domain. The reciprocal influence of the nucleotide state on microtubule binding and the microtubule on nucleotide binding and hydrolysis leads to coupling between the chemical and mechanical cycles, driving the operation of these nanomachines. As in the myosin field, a major goal in the kinesin field is to characterize the functional properties of the different nucleotide states and the kinetics of the transitions between them.

**Fig. 1:**
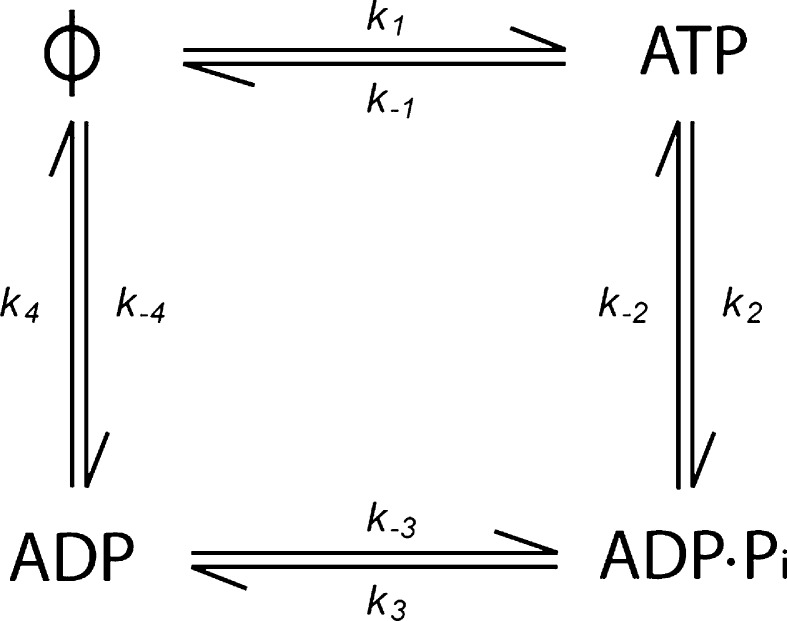
The minimal ATP turnover cycle for an ATP hydrolysing enzyme such as a kinesin. There are four possible nucleotide states: nucleotide free (ϕ), ATP-bound, ADP–P_i_-bound, and ADP-bound

Despite the high sequence conservation of motor domains between different sub-families of kinesin, typically 50 % sequence identity, there is a remarkable diversity of kinetic and structural properties of the nucleotide-gated switch. This diversity accounts, to a large extent, for the remarkable variety of biological functions performed by the kinesin superfamily. Kinesins fall into several major subfamilies, denoted kinesin-1 to kinesin-14, as well as many orphan kinesins (Kinesin Home Page; Lawrence et al. 2004; Endow et al. 2010). The cell biological functions of kinesins include the translocation of organelles such as membrane-bounded vesicles (kinesin-1 and -3; Hirokawa et al. 2009), RNA particles (kinesin-1; Kanai et al. 2004; Gaspar 2011), intraflagellar transport particles (kinesin-2; Cole et al. 1998; Ou et al. 2005), and chromosomes (kinesin-4 and -7; Mazumdar and Misteli 2005); microtubule sliding (kinesin-5 and -14; Peterman and Scholey 2009), microtubule depolymerisation (kinesin-8 and -13; Howard and Hyman 2007; Wordeman 2005, and the kinesin-14 Kar3; Huyett et al. 1998; Saunders et al. 1997) or microtubule nucleation/elongation (the kinesin-7s, Kip2p and CENP-E; Huyett et al. 1998; Sardar et al. 2010, and the kinesin-10 NOD; Cui et al. 2005). In this review, we discuss the ATP turnover cycle (Fig. 1) of different members of the kinesin superfamily and show how differences in the kinetics of the cycle, together with variation in the properties of the different nucleotide states, can account for many of the observed functions of different kinesins.

## Translocating and microtubule-regulating kinesins

Members of the kinesin superfamily studied to date fall into one or both of two major classes: translocating kinesins that move with respect to the microtubule lattice (e.g. kinesin-1, -2, -3, -4, -5, -7, -8, -14); and microtubule-regulating kinesins that bind to microtubule ends and regulate polymerization or depolymerization (e.g. kinesin-7, -8, -10, -13). The translocating kinesins can in turn be divided into two classes: highly processive motors (e.g. kinesin-1, -7, -8) that individually can take many steps along a microtubule before dissociating (i.e. long run lengths), and less processive motors (e.g. kinesin-3, -5, -14) that take only one or a few of steps along the lattice before dissociating. The microtubule-regulating kinesins can in turn be divided into depolymerases (kinesin-8, -13 and perhaps -14) that antagonize growth, assemblers (the kinesin-7s CENP-E and Kip2 and the kinesin-10 NOD) that promote growth, and inhibitors that antagonize both growth and disassembly (kinesin-8). Table 1 summarizes the main functional classes of kinesins.

**Table 1:** Functional properties of kinesin sub-families

Kinesin subfamily	Examples	Translocase	Processive	Sliding	Microtubule regulation
Kinesin-1	KHC (Coy et al. 1999), Kif5 (Kanai et al. 2000)	Yes (+end)	Yes	No	No
Kinesin-2	Fla10 (Kozminski et al. 1995)	Yes (+end)	Yes	No	No
Kinesin-3	Kif1 (Okada and Hirokawa 1999)	Yes (+end)	no	No	No
Kinesin-4	Kif4 (Sekine et al. 1994)	Yes (+end)	Yes	Yes (anti-||)	Depolymerisation, +end
Kinesin-5	Eg5 (Sawin et al. 1992)	Yes (+end)	no	Yes (anti-||)	Deploymerisation (+end)
Kinesin-7	CENP-E (Wood et al. 1997)	Yes (+end)	Yes	No	Elongation
Kinesin-8	Kip3 (Varga et al. 2006)	Yes (+end)	Yes	No	Depolymerisation (+end)
Kinesin-10	NOD (Afshar et al. 1995)	No	N/a	No	Polymerization (+end)
Kinesin-13	MCAK (Wordeman and Mitchison 1995)	No (diffusive)	N/a	No	Depolymerisation (+ & −end)
Kinesin-14	NCD (Chandra et al. 1993)	Yes (−end)	No	Yes (anti-||)	Depolymerisation (−end)

**Table 2:** Microtubule-stimulated ATPase and translocation speeds for selected kinesins

Protein	Kinesin family	MT stimulated ATPase (s^−1^)	Velocity (nm s^−1^)	Source
*D. melanogaster* KHC	1	44	770	Coy et al. (1999)
*R. rattus* Kif5c	1	33	460	McVicker et al. (2011)
*H. sapiens* ‘cys-light’ KHC	1	31	420	Yildiz et al. (2008)
*M. musculus* Kif1a	3	110	140	Okada and Hirokawa (1999)
*N. crassa* NcKin3	3	*23* ^a^	590	Adio et al. (2006)
*A. thaliana* FRA1	4	5.9^a^	400	Zhu and Dixit (2011)
*X. laevis* Eg5	5	*1.9*	63	Lockhart and Cross (1996)
*X. laevis* CENP-E	7	12^a^	340	Yardimci et al. (2008), Rosenfeld et al. (2009)
*S. cerevisiae* Kip3p	8	1.8	12	Gupta et al. (2006)
*D. melanogaster* NCD	14	*2.3* ^a^	140	Case et al. (1997), Shimizu et al. (1995)
*S. pombe* Klp2	14	*3.8* ^a^	42	Braun et al. (2009)
*S. pombe* Plk1	14	*2.0*	42	Furuta et al. (2008)

^a^The ATPase activity was not measured under the same conditions as the motility and may be underestimated. Italicized values indicate non-processive motors

## The ATP turnover mechanisms of processive translocating kinesins

The founding and most intensively studied kinesin subfamily is kinesin-1 (conventional kinesin). Members of the kinesin 1 family are the classic cargo carriers able to take many steps along the microtubule (Howard et al. 1989; Svoboda et al. 1993), even when working against an elastic (Svoboda and Block 1994; Meyhofer and Howard 1995; Gittes et al. 1996) or viscous load (Hunt et al. 1994). The main characteristic of the ATP turnover cycle of the kinesin 1 family is that it is limited by ADP dissociation (Fig. 1, *k*_4_), which is very slow in the absence of microtubules and accelerated approximately 5000-fold by microtubules (Hackney 1988). Through accelerating ADP release, microtubules increase the ATPase rate from 0.01 s^−1^ to approximately 50 s^−1^ (Kuznetsov and Gelfand 1986; Hackney and Stock 2008).

The ATP turnover cycles of other highly processive kinesin subfamilies have not been studied in such detail as the kinesin-1s, so it is not possible to say that these are the definitive characteristics of a processive translocase. However, several other processive kinesins have been characterized to a lesser extent and fit with this basic framework. The ATPase cycle of the mitotic kinesin CENP-E, a member of the kinesin 7 family, has been studied in the presence of microtubules (Rosenfeld et al. 2009). The basal ATPase cycle has yet to be determined, therefore it is unknown if it possesses the same rate limiting step of ADP dissociation characteristic of other translocating kinesins. It is also unknown to what degree microtubules stimulate the basal ATPase. However, information from the ATP turnover cycle in the presence of microtubules can shed light on the processivity of a kinesin. The rate constant for the bimolecular association of the kinesin with the microtubule can be estimated by dividing the microtubule-stimulated ATPase (also termed V_max_ or *k*_cat_) by the concentration of microtubules at which the ATPase is half saturated (K_0.5, MT_). This value assumes that only 1 ATP is hydrolysed per encounter with the microtubule (Hackney 1995a). By comparing this value with either a theoretical limit for such a bimolecular association or a measured value obtained by detecting each productive encounter of a kinesin with the microtubule by monitoring the stimulated release of ADP (*k*_bi_) (Hackney 1995a), it is possible to determine if the assumption of 1 ATP hydrolysed per microtubule encounter is correct. In the case of kinesin-1, the ratio of *k*_cat_/K_0.5, MT_ to *k*_bi_ suggests that ~120 ATPs are hydrolysed per encounter of a motor with the microtubule; this is consistent with the high processivity of the kinesin-1 family (Hackney 1995a). For CENP-E, *k*_cat_/K_0.5, MT_ = 134 μM^−1^s^−1^; because this is fivefold larger than the theoretical limit for protein-protein binding (Hackney 1995b) these data suggest that CENP-E produces multiple cycles of ATP turnover per microtubule encounter. If each turnover results in a step, then there are many steps per microtubule encounter, indicating processive movement (Rosenfeld et al. 2009).

One of the most highly processive translocating kinesins is the kinesin-8 family member from *S. cerevisiae*, Kip3p (Varga et al. 2006). The full cycle of this kinesin has not been determined in the absence or presence of microtubules and so the rate-limiting step of the basal cycle is not known. However, the degree of stimulation of the basal ATPase by microtubules is much less that that for kinesin-1: only a little over tenfold compared to the 5000-fold stimulation seen for kinesin-1 (Gupta et al. 2006). Also, the ratio of *k*_cat_/K_0.5, MT_ is small (0.7 μM^−1^s^−1^ (Gupta et al. 2006)): more than 40-fold lower than the theoretical limit for the bimolecular association of a molecule the size of kinesin (Hackney 1995b) and more than threefold lower than the measured value for a kinesin-1 (Hackney 1995a). A possible explanation is that the ATPase measurements are done under much lower ionic strength buffer conditions compared to the motility assays: the motility is very dependent on the ionic strength, and in particular the on-rate increases strongly as the ionic strength is increased (Varga et al. 2009).

**Fig. 2:**
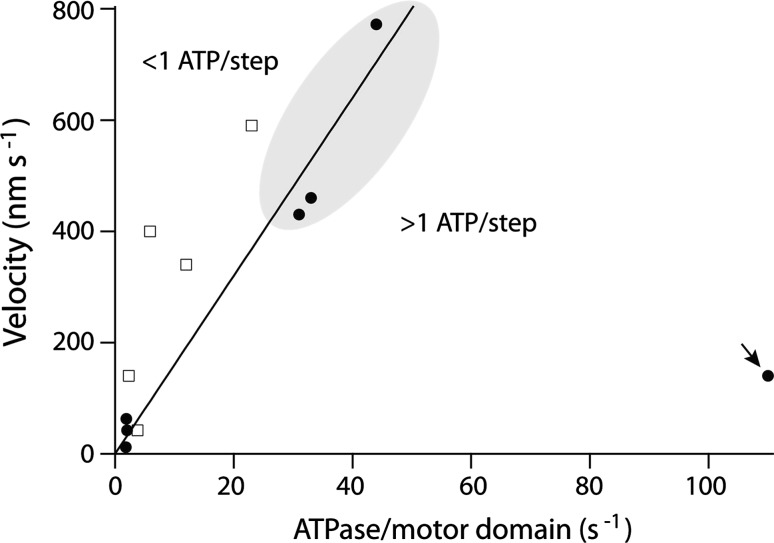
The microtubule stimulated ATPase for selected kinesins plotted against the measured velocity for these kinesins determined from either stepping or gliding assays (data from Table 2). The *closed symbols* denote data in which both assays were performed under identical or near-identical conditions. The *open symbols* denote data in which the assays are performed under different conditions, and the ATPase may not be fully activated. The *solid line* corresponds to 1:1 coupling between the ATPase and stepping velocity with a step size of 8 nm (Eq. 1). *Highlighted* region indicates data for kinesin-1. The *arrow* indicates data for the monomeric kinesin, Kif1a

Even a single-headed kinesin can exhibit high processivity. The kinesin-3, Kif1a, has a mean run length equivalent to ~100 steps along the microtubule lattice (Okada and Hirokawa 1999). This processivity is not governed by the walking model common to 2-headed translocating kinesins, in which one head anchors the kinesin to the microtubule whilst the other takes a step, but relies on the weakly bound state maintaining the single head on the lattice long enough to allow it to find the next binding site (Okada and Hirokawa 2000). This mode of action is reflected in the relationship between the ATPase of Kif1a and its translocation velocity (Fig. 2, arrow). Kif1a hydrolyses many ATPs for each forward step reflecting the low degree of coupling between stepping and ATP turnover in this single-headed system.

## Low-processivity translocating kinesins

The best studied of this group are the mitotic kinesins of the kinesin-5 and kinesin-14 families. Members of these families have been shown the have broadly similar ATP turnover cycles to that of the highly processive kinesin-1 family. The kinesin-5 motor Eg5 and the kinesin-14 motor NCD both have basal turnover cycles limited by ADP dissociation and which are stimulated many hundred-fold by microtubules (~300-fold and ~1000-fold for Eg5 and NCD, respectively) (Lockhart and Cross 1996; Cochran et al. 2004; Lockhart and Cross 1994; Pechatnikova and Taylor 1997). It is in the finer details of the ratio of the various rate constants associated with the chemical and mechanical cycles that the explanation for their low processivity is found. For NCD the ratio of the microtubule-stimulated ATPase to the rate constant for dissociation of NCD from the microtubule upon binding nucleotide is 0.3 (Pechatnikova and Taylor 1997). Thus, NCD turns over on average 0.3 ATP molecules before dissociation from the microtubule, implying that NCD tends to dissociate from the microtubule before completing the ATP turnover cycle and this is reflected in low processivity of translocation.

## Relationship between ATPase rate and velocity of translocation

The kinesin-1 step size is 8-nm (Svoboda et al. 1993; Carter and Cross 2005), corresponding to a single binding site per tubulin dimmer (Ray et al. 1993; Harrison et al. 1993). We expect that all the processive kinesins will have 8-nm steps, so if stepping is tightly coupled to ATP turnover, we expect that the speed will be

1 $$ v = 2 \times 8 \times k_{\text{cat}} ({\text{nm}}/{\text{s}}) $$

where *k*_cat_ is the ATPase rate per motor domain and the two is due to processive motors having two motor domains. If the motor is weakly coupled we might expect multiple ATPs hydrolysed per step. The alternative, that there are many steps per ATP has been ruled out in the case of kinesin-1 as single-molecule tracking shows that there are no bursts of steps at low ATP concentrations (Hua et al. 1997; Schnitzer and Block 1997). Figure 2 shows translocation velocity plotted against the ATPase for several kinesins. There is a strong correlation between speed and ATPase, consistent with coupling between the chemical and mechanical cycles. A similar correlation is seen with myosin motors: myosin-II isoforms from faster muscles have higher ATPase rates (Barany 1967). In the case of kinesins, most points fall above or close to the line corresponding to one step per ATP hydrolysed (Eq. 1), consistent with at least one ATP being required per 8 nm step. Only the single-headed kinesin, Kif1a, hydrolyses many ATP per step (arrow in Fig. 2). For the best-studied motor, kinesin-1, in which both velocity and ATPase have been measured under almost identical conditions, there is very close agreement with Eq. 1, indicating that there is a tight coupling of one ATP per step (highlighted in Fig. 2).

## Microtubule-regulating kinesins

**Fig. 3:**
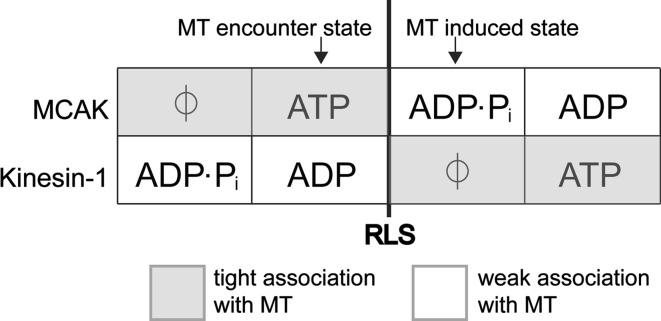
Comparison of the association with the microtubule (MT) of the non-motile kinesin MCAK and the translocating kinesin-1 according to nucleotide state. The ATP turnover cycles are aligned with respect to the rate-limiting step (RLS) in the absence of microtubules (*bold line*). The kinesin will meet the filament predominantly in the nucleotide state prior to the RLS. Interaction with the filament accelerates the RLS driving the motor protein to the next nucleotide state in the cycle. The change in affinity triggered by the microtubule-stimulated change in nucleotide state is reversed for MCAK compared with kinesin-1

Members of the kinesin-10 and kinesin-13 families do not function as the majority of other kinesins. They have no translocating activity but instead interact with the microtubule lattice in a diffusive manner, remaining in a weakly bound state rather than cycling through alternate weak and strong binding states as do the translocating kinesins (Cui et al. 2005; Hunter et al. 2003; Helenius et al. 2006; Cochran et al. 2009). The only members of these families to have their ATP turnover cycles studied in detail are the kinesin-10, NOD and the kinesin-13, MCAK. Both these kinesins have been found to have atypical basal ATPase cycles, in that the rate-limiting step is the cleavage of ATP (Fig. 1, *k*_2_) rather than ADP dissociation (Fig. 1, *k*_4_) (Cochran et al. 2009; Friel and Howard 2011). For both MCAK and NOD this results in the motor domain predominantly meeting the microtubule lattice in an ATP-containing state rather the ADP containing state in which translocating motors predominantly exist in solution. For MCAK, this implies that the motor domain initially binds tightly to the microtubule lattice, as the ATP state is a strong-binding rigor state (Helenius et al. 2006). After interaction with the microtubule, ATP is rapidly hydrolysed and the MCAK motor domain enters one of the weakly microtubule bound nucleotide states, ADP·P_i_ or ADP. This is the reverse of the initial interaction with the microtubule of a translocating kinesin such as kinesin-1 (Fig. 3). In the weakly bound nucleotide states MCAK diffuses on the microtubule lattice, until it either detaches from the microtubule or reaches the microtubule end. The microtubule end accelerates the dissociation of ADP, stimulating a nucleotide exchange of ADP for ATP. This results in tight binding of the ATP containing motor domain, facilitating tubulin dissociation from the microtubule end (Moores et al. 2003; Hunter et al. 2003). In this way MCAK’s ATP turnover cycle is tuned to enable this kinesin to identify and bind to microtubule ends where it can effect its activity as a depolymerase. The ATPase rate for MCAK is stimulated ~5000-fold by the presence of microtubules, the microtubule in general accelerates the ATP cleavage step and the microtubule end specifically further stimulates the ATPase by accelerating ADP dissociation (Friel and Howard 2011).

Rate-limiting ATP cleavage in the basal ATPase cycle may be a characteristic feature of non-motile kinesins. This step is also rate limiting in the basal ATP turnover cycle of the kinesin-10 NOD (Cochran et al. 2009). However, how this atypical ATP turnover cycle adapts NOD for its function of maintaining attachment to the microtubule plus end remains unclear. The ATPase rate of the NOD motor domain has been measured in the presence and absence of microtubules. However, the effect of microtubules on ATP turnover by the isolated motor domain is unresolved. One study indicates that the microtubule stimulates the ATPase rate for NOD by more than 2000-fold (Matthies et al. 2001); whilst a second study indicates that microtubules do not stimulate the ATPase activity of NOD (Cochran et al. 2009). It has been suggested that NOD tracks microtubule ends by having a different response to the microtubule end than to the lattice (Cochran et al. 2009), in the same way as determined for MCAK (Friel and Howard 2011). However, no evidence as yet exists to show that this is the case. Nevertheless, rate-limiting ATP cleavage in the basal ATPase cycle combined with microtubule end-specific stimulation of step/s in the cycle may be characteristic features of non-motile, microtubule-end-tracking kinesins.

Members of the kinesin superfamily perform a variety of microtubule-based functions, including different speeds of translocation, different degrees of processivity, tracking the microtubule end and regulation of microtubule dynamics. Variation in the ATP turnover cycle amongst superfamily members provides one way in which the characteristic motor domain can be tuned to different functions. Dissection of the cycle of ATP turnover of a kinesin, both in the presence and absence of microtubules, can help explain the particular behaviour observed. The precise nature of the way in which a kinesin utilises ATP must be known as well as the nature of its interaction with the microtubule to fully comprehend the action of an individual kinesin.

## Definitions

ATPase: Molecules of ATP hydrolysed per motor domain per second
Basal ATPase: ATPase in the absence of microtubules or unpolymerised tubulin
Microtubule stimulated ATPase: Maximum ATPase (V_max_) at saturating concentrations of polymerised tubulin

## References

[CR1] Adio S, Bloemink M, Hartel M, Leier S, Geeves MA, Woehlke G (2006). Kinetic and mechanistic basis of the nonprocessive Kinesin-3 motor NcKin3. J Biol Chem.

[CR2] Afshar K, Barton NR, Hawley RS, Goldstein LS (1995). DNA binding and meiotic chromosomal localization of the Drosophila nod kinesin-like protein. Cell.

[CR3] Barany M (1967) ATPase activity of myosin correlated with speed of muscle shortening. J Gen Physiol 50 (6):Suppl:197–218 10.1085/jgp.50.6.197 PMC2225740 4227924

[CR4] Braun M, Drummond DR, Cross RA, McAinsh AD (2009). The kinesin-14 Klp2 organizes microtubules into parallel bundles by an ATP-dependent sorting mechanism. Nat Cell Biol.

[CR5] Carter NJ, Cross RA (2005). Mechanics of the kinesin step. Nature.

[CR6] Case RB, Pierce DW, Hom-Booher N, Hart CL, Vale RD (1997). The directional preference of kinesin motors is specified by an element outside of the motor catalytic domain. Cell.

[CR7] Chandra R, Salmon ED, Erickson HP, Lockhart A, Endow SA (1993). Structural and functional domains of the Drosophila ncd microtubule motor protein. J Biol Chem.

[CR8] Cochran JC, Sontag CA, Maliga Z, Kapoor TM, Correia JJ, Gilbert SP (2004). Mechanistic analysis of the mitotic kinesin Eg5. J Biol Chem.

[CR9] Cochran JC, Sindelar CV, Mulko NK, Collins KA, Kong SE, Hawley RS, Kull FJ (2009). ATPase cycle of the nonmotile kinesin NOD allows microtubule end tracking and drives chromosome movement. Cell.

[CR10] Cole DG, Diener DR, Himelblau AL, Beech PL, Fuster JC, Rosenbaum JL (1998). Chlamydomonas kinesin-II-dependent intraflagellar transport (IFT): IFT particles contain proteins required for ciliary assembly in Caenorhabditis elegans sensory neurons. J Cell Biol.

[CR11] Coy DL, Wagenbach M, Howard J (1999). Kinesin takes one 8-nm step for each ATP that it hydrolyzes. J Biol Chem.

[CR12] Crevel I, Carter N, Schliwa M, Cross R (1999). Coupled chemical and mechanical reaction steps in a processive Neurospora kinesin. EMBO J.

[CR13] Cui W, Sproul LR, Gustafson SM, Matthies HJ, Gilbert SP, Hawley RS (2005). Drosophila Nod protein binds preferentially to the plus ends of microtubules and promotes microtubule polymerization in vitro. Mol Biol Cell.

[CR14] Endow SA, Kull FJ, Liu H (2010). Kinesins at a glance. J Cell Sci.

[CR15] Friel CT, Howard J (2011). The kinesin-13 MCAK has an unconventional ATPase cycle adapted for microtubule depolymerization. EMBO J.

[CR16] Furuta K, Edamatsu M, Maeda Y, Toyoshima YY (2008). Diffusion and directed movement: in vitro motile properties of fission yeast kinesin-14 Pkl1. J Biol Chem.

[CR17] Gaspar I (2011) Microtubule-based motor-mediated mRNA localization in Drosophila oocytes and embryos. Biochem Soc Trans 39(5):1197–1201 10.1042/BST0391197 21936788

[CR18] Gittes F, Meyhofer E, Baek S, Howard J (1996). Directional loading of the kinesin motor molecule as it buckles a microtubule. Biophys J.

[CR19] Gupta ML, Carvalho P, Roof DM, Pellman D (2006). Plus end-specific depolymerase activity of Kip3, a kinesin-8 protein, explains its role in positioning the yeast mitotic spindle. Nat Cell Biol.

[CR20] Hackney DD (1988). Kinesin ATPase: rate-limiting ADP release. Proc Natl Acad Sci USA.

[CR21] Hackney DD (1994). Evidence for alternating head catalysis by kinesin during microtubule-stimulated ATP hydrolysis. Proc Natl Acad Sci USA.

[CR22] Hackney DD (1995). Highly processive microtubule-stimulated ATP hydrolysis by dimeric kinesin head domains. Nature.

[CR23] Hackney DD (1995b) Implications of diffusion-controlled limit for processivity of dimeric kinesin head domains. Biophys J 68(4 Suppl):267S–269S; discussion 269S–270S PMC1281942 7787088

[CR24] Hackney DD, Stock MF (2008). Kinesin tail domains and Mg^2+^ directly inhibit release of ADP from head domains in the absence of microtubules. Biochemistry.

[CR25] Harrison BC, Marchese-Ragona SP, Gilbert SP, Cheng N, Steven AC, Johnson KA (1993). Decoration of the microtubule surface by one kinesin head per tubulin heterodimer. Nature.

[CR26] Helenius J, Brouhard G, Kalaidzidis Y, Diez S, Howard J (2006). The depolymerizing kinesin MCAK uses lattice diffusion to rapidly target microtubule ends. Nature.

[CR27] Hirokawa N, Noda Y, Tanaka Y, Niwa S (2009). Kinesin superfamily motor proteins and intracellular transport. Nat Rev Mol Cell Biol.

[CR28] Hirose K, Akimaru E, Akiba T, Endow SA, Amos LA (2006). Large conformational changes in a kinesin motor catalyzed by interaction with microtubules. Mol Cell.

[CR29] Howard J, Hyman AA (2007). Microtubule polymerases and depolymerases. Curr Opin Cell Biol.

[CR30] Howard J, Hudspeth AJ, Vale RD (1989). Movement of microtubules by single kinesin molecules. Nature.

[CR31] Hua W, Young EC, Fleming ML, Gelles J (1997). Coupling of kinesin steps to ATP hydrolysis. Nature.

[CR32] Hunt AJ, Gittes F, Howard J (1994). The force exerted by a single kinesin molecule against a viscous load. Biophys J.

[CR33] Hunter AW, Caplow M, Coy DL, Hancock WO, Diez S, Wordeman L, Howard J (2003). The kinesin-related protein MCAK is a microtubule depolymerase that forms an ATP-hydrolyzing complex at microtubule ends. Mol Cell.

[CR34] Huyett A, Kahana J, Silver P, Zeng X, Saunders WS (1998). The Kar3p and Kip2p motors function antagonistically at the spindle poles to influence cytoplasmic microtubule numbers. J Cell Sci.

[CR35] Kanai Y, Okada Y, Tanaka Y, Harada A, Terada S, Hirokawa N (2000). KIF5C, a novel neuronal kinesin enriched in motor neurons. J Neurosci.

[CR36] Kanai Y, Dohmae N, Hirokawa N (2004). Kinesin transports RNA: isolation and characterization of an RNA-transporting granule. Neuron.

[CR37] Kikkawa M, Sablin EP, Okada Y, Yajima H, Fletterick RJ, Hirokawa N (2001). Switch-based mechanism of kinesin motors. Nature.

[CR70] Kinesin Home Page http://www.cellbio.duke.edu/kinesin/

[CR38] Kozminski KG, Beech PL, Rosenbaum JL (1995). The Chlamydomonas kinesin-like protein FLA10 is involved in motility associated with the flagellar membrane. J Cell Biol.

[CR39] Kuznetsov SA, Gelfand VI (1986). Bovine brain kinesin is a microtubule-activated ATPase. Proc Natl Acad Sci USA.

[CR40] Lawrence CJ, Dawe RK, Christie KR, Cleveland DW, Dawson SC, Endow SA, Goldstein LS, Goodson HV, Hirokawa N, Howard J, Malmberg RL, McIntosh JR, Miki H, Mitchison TJ, Okada Y, Reddy AS, Saxton WM, Schliwa M, Scholey JM, Vale RD, Walczak CE, Wordeman L (2004). A standardized kinesin nomenclature. J Cell Biol.

[CR41] Lockhart A, Cross RA (1994). Origins of reversed directionality in the ncd molecular motor. EMBO J.

[CR42] Lockhart A, Cross RA (1996). Kinetics and motility of the Eg5 microtubule motor. Biochemistry.

[CR43] Ma YZ, Taylor EW (1997). Interacting head mechanism of microtubule-kinesin ATPase. J Biol Chem.

[CR44] Marx A, Hoenger A, Mandelkow E (2009). Structures of kinesin motor proteins. Cell Motil Cytoskeleton.

[CR45] Matthies HJ, Baskin RJ, Hawley RS (2001). Orphan kinesin NOD lacks motile properties but does possess a microtubule-stimulated ATPase activity. Mol Biol Cell.

[CR80] Mazumdar M, Misteli T (2005) Chromokinesins: multitalented players in mitosis. Trends Cell Biol 15(7):349–355 10.1016/j.tcb.2005.05.006 15946846

[CR46] McVicker DP, Chrin LR, Berger CL (2011). The nucleotide-binding state of microtubules modulates kinesin processivity and the ability of Tau to inhibit kinesin-mediated transport. J Biol Chem.

[CR47] Meyhofer E, Howard J (1995). The force generated by a single kinesin molecule against an elastic load. Proc Natl Acad Sci USA.

[CR48] Miki H, Okada Y, Hirokawa N (2005). Analysis of the kinesin superfamily: insights into structure and function. Trends Cell Biol.

[CR49] Moores CA, Hekmat-Nejad M, Sakowicz R, Milligan RA (2003). Regulation of KinI kinesin ATPase activity by binding to the microtubule lattice. J Cell Biol.

[CR50] Okada Y, Hirokawa N (1999). A processive single-headed motor: kinesin superfamily protein KIF1A. Science.

[CR51] Okada Y, Hirokawa N (2000). Mechanism of the single-headed processivity: diffusional anchoring between the K-loop of kinesin and the C terminus of tubulin. Proc Natl Acad Sci USA.

[CR52] Ou G, Blacque OE, Snow JJ, Leroux MR, Scholey JM (2005). Functional coordination of intraflagellar transport motors. Nature.

[CR53] Pechatnikova E, Taylor EW (1997). Kinetic mechanism of monomeric non-claret disjunctional protein (Ncd) ATPase. J Biol Chem.

[CR54] Peterman EJ, Scholey JM (2009). Mitotic microtubule crosslinkers: insights from mechanistic studies. Curr Biol.

[CR55] Ray S, Meyhofer E, Milligan RA, Howard J (1993). Kinesin follows the microtubule’s protofilament axis. J Cell Biol.

[CR56] Rosenfeld SS, van Duffelen M, Behnke-Parks WM, Beadle C, Corrreia J, Xing J (2009). The ATPase cycle of the mitotic motor CENP-E. J Biol Chem.

[CR57] Sack S, Kull FJ, Mandelkow E (1999). Motor proteins of the kinesin family. Structures, variations, and nucleotide binding sites. Eur J Biochem.

[CR58] Sardar HS, Luczak VG, Lopez MM, Lister BC, Gilbert SP (2010). Mitotic kinesin CENP-E promotes microtubule plus-end elongation. Curr Biol.

[CR59] Saunders W, Hornack D, Lengyel V, Deng C (1997). The Saccharomyces cerevisiae kinesin-related motor Kar3p acts at preanaphase spindle poles to limit the number and length of cytoplasmic microtubules. J Cell Biol.

[CR60] Sawin KE, LeGuellec K, Philippe M, Mitchison TJ (1992). Mitotic spindle organization by a plus-end-directed microtubule motor. Nature.

[CR61] Schnitzer MJ, Block SM (1997). Kinesin hydrolyses one ATP per 8-nm step. Nature.

[CR62] Sekine Y, Okada Y, Noda Y, Kondo S, Aizawa H, Takemura R, Hirokawa N (1994). A novel microtubule-based motor protein (KIF4) for organelle transports, whose expression is regulated developmentally. J Cell Biol.

[CR63] Shimizu T, Sablin E, Vale RD, Fletterick R, Pechatnikova E, Taylor EW (1995). Expression, purification, ATPase properties, and microtubule-binding properties of the ncd motor domain. Biochemistry.

[CR64] Svoboda K, Block SM (1994). Force and velocity measured for single kinesin molecules. Cell.

[CR65] Svoboda K, Schmidt CF, Schnapp BJ, Block SM (1993). Direct observation of kinesin stepping by optical trapping interferometry. Nature.

[CR66] Varga V, Helenius J, Tanaka K, Hyman AA, Tanaka TU, Howard J (2006). Yeast kinesin-8 depolymerizes microtubules in a length-dependent manner. Nat Cell Biol.

[CR81] Varga V, Leduc C, Bormuth V, Diez S, Howard J (2009) Kinesin-8 motors act cooperatively to mediate length-dependent microtubule depolymerization. Cell 138(6):1174–1183 10.1016/j.cell.2009.07.032 19766569

[CR67] Wood KW, Sakowicz R, Goldstein LS, Cleveland DW (1997). CENP-E is a plus end-directed kinetochore motor required for metaphase chromosome alignment. Cell.

[CR68] Wordeman L (2005). Microtubule-depolymerizing kinesins. Curr Opin Cell Biol.

[CR69] Wordeman L, Mitchison TJ (1995). Identification and partial characterization of mitotic centromere-associated kinesin, a kinesin-related protein that associates with centromeres during mitosis. J Cell Biol.

[CR71] Yardimci H, van Duffelen M, Mao Y, Rosenfeld SS, Selvin PR (2008). The mitotic kinesin CENP-E is a processive transport motor. Proc Natl Acad Sci USA.

[CR72] Yildiz A, Tomishige M, Gennerich A, Vale RD (2008). Intramolecular strain coordinates kinesin stepping behavior along microtubules. Cell.

[CR73] Yun M, Zhang X, Park CG, Park HW, Endow SA (2001). A structural pathway for activation of the kinesin motor ATPase. EMBO J.

[CR74] Zhu C, Dixit R (2011). Single molecule analysis of the Arabidopsis FRA1 kinesin shows that it is a functional motor protein with unusually high processivity. Mol Plant.

